# Comparison of ceramic-on-ceramic to metal-on-polyethylene bearing surfaces in total hip arthroplasty: a meta-analysis of randomized controlled trials

**DOI:** 10.1186/s13018-015-0163-2

**Published:** 2015-02-03

**Authors:** Dongcai Hu, Kai Tie, Xiao Yang, Yang Tan, Mohammed Alaidaros, Liaobin Chen

**Affiliations:** Department of Orthopaedic Surgery, Zhongnan Hospital of Wuhan University, Wuhan, Hubei 430071 China; Department of Intensive Care Unit, Zhongnan Hospital of Wuhan University, Wuhan, Hubei 430071 China

**Keywords:** Total hip arthroplasty, Bearing surface, Ceramic-on-ceramic, Metal-on-polyethylene, Meta-analysis

## Abstract

**Background:**

In recent years, the choice of ceramic-on-ceramic (COC) and metal-on-polyethylene (MOP) in primary total hip arthroplasty (THA) remains controversial. The purpose of this study was to compare the reliability and durability of COC with that of MOP bearing surfaces in THA.

**Methods:**

Based on prospective randomized controlled trials (RCTs) searched from Pubmed, Embase, Web of Science, and Cochrane central database, we performed a meta-analysis for comparing clinical and radiographic outcomes of COC with those of MOP. Two investigators independently selected studies, extracted data, and assessed risk of bias. Relative risks and weighted mean differences from each trial were pooled using random-effect or fixed-effect models depending on the heterogeneity of the included studies.

**Results:**

Five RCTs involving 897 patients with 974 hips met predetermined inclusion criteria. Our results demonstrated COC significantly decreased the risks of revision, osteolysis and radiolucent line, aseptic loosening, and dislocation and increased the risks of squeaking and intraoperative implant fracture compared with MOP. There was no significant difference between the two groups in postoperative hip function, deep infection, and heterotopic ossification.

**Conclusions:**

Generally, despite more squeaking and intraoperative implant fracture, our findings support the use of COC bearing surface which has lower rates of revision, osteolysis and radiolucent line, aseptic loosening, and dislocation compared with MOP.

## Background

Total hip arthroplasty (THA) has become a common treatment for end-stage hip joint diseases. With improved implant designs and surgical techniques, bearing surface wear, and the resultant wear-induced osteolysis have become a major limitation to prosthetic long-term survivorship, particularly in young and active patients [1,2].

Metal-on-polyethylene (MOP) bearing surface, a bearing surface with good long-term results in elderly patients, once was taken as gold standard for THA [3,4]. But in the past decades, it became clear that polyethylene liner wear debris generated with time was associated with the occurrence of osteolysis which leads to subsequent loosening and eventual implant failure. It has been reported that the osteolysis rate of MOP is as high as 26%, and aseptic loosening rate is 3% at 10-year follow-up [1]. In an attempt to avoid the problems caused by polyethylene wear debris, ceramic-on-ceramic (COC) bearing surface has been developed as an alternative. COC offered several theoretical advantages, such as extreme hardness and scratch resistance, which improved lubrication that creates low coefficient of friction resulting in excellent wear resistance and low osteolysis rate [5,6]. Hence, COC bearing surfaces are increasingly used for THA. Literature reported that none of the 144 hips with COC bearing surfaces occurred osteolysis at 10-year follow-up [1]. However, concerns still exist about its high cost and adverse events, such as the difficulty in inserting the ceramic liner into the acetabular cup, ceramic fracture, and audible squeaking [1,4-6]. Therefore, clinically MOP and COC, which is the better bearing surface is the question and option the surgeons have to face and choose. To clarify this, some randomized controlled trials (RCTs) were conducted to compare COC and MOP in search for the optimal bearing surface [1,3-18]. But different studies attained variant conclusions. Some reported that COC was superior to MOP in clinical outcomes [4,5] while the others reported that there were no statistically significant differences between the COC and MOP [6,7,17].

Although one previous meta-analysis has been published [2], the data presented are not convincing: it is largely based upon observational non-randomized or non-controlled studies, it follows a random effects model due to substantial unsolvable heterogeneity, and the *P*-value is marginal (*P* = 0.05). Under the circumstance, it is obvious that a more objective, reliable, and persuasive data is needed to reevaluate the two bearing surfaces.

The objective of the current study was to compare the hip function, prosthetic survivorship, and related complications of COC and MOP bearing surfaces in THA by performing a new meta-analysis which only included the published RCTs.

## Materials and methods

### Search strategy

Two trained investigators independently searched the online databases including Pubmed, Embase, Web of Science, and Cochrane central database (all to March 23, 2014). The search terms were ((((((total hip replacement) OR total hip arthroplasty)) AND polyethylene) AND metal) AND ceramic) AND random*.

### Eligibility criteria

Articles that met the following criteria were included: (1) target population: individuals underwent primary THA; (2) intervention: a comparison between COC and MOP bearing surfaces; (3) outcome: studies that reported hip function, complications, or radiographic outcomes of THA (at least one desirable outcome); (4) methodological criterion: prospective, RCTs; and (5) full text was published in English.

### Study identification

Two trained investigators independently identified studies using the above eligibility criteria. If the title or the abstract was judged to be potentially eligible, the full-text article was reviewed. Any conflict was resolved by discussion with the third investigator.

### Assessment of study quality

The quality of each study was independently assessed by two of trained investigators with use of Physiotherapy Evidence Database (PEDro) scale [19,20]. The PEDro scale was a widely used checklist which included 11 items. Each item was scored “yes” or “no” with a maximum score of 10 because the first item was not scored. Any trial with a score of 6 or more was considered high quality. Any disagreement was resolved by discussion with the third investigator.

### Data extraction

Relevant data was extracted independently by two trained investigators. Information retrieved from each study included the following items: demographic information, Harris hip score (HHS), number of participants and hips allocated to each group, number of revision, number of osteolysis and radiolucent line, number of aseptic loosening, number of squeaking, number of intraoperative and postoperative implant fracture, number of dislocation, number of deep infection, and number of heterotopic ossification. Any disagreement was discussed with the third investigator and resolved by consensus.

### Statistical analysis

The outcomes for measure included HHS, revision, osteolysis and radiolucent line, aseptic loosening, squeaking, intraoperative and postoperative implant fracture, dislocation, deep infection and heterotopic ossification.

RevMan 5.1 software was used for data analysis. For each study, relative risks (RR) and 95% confidence intervals (CI) were calculated for dichotomous outcomes. Mean difference (MD) and 95% CI were calculated for continuous outcome. The significance level was defined as *P* < 0.05. Heterogeneity between comparable studies was tested with the use of chi-square (*χ*^2^) and *I*^2^ test. *P* > 0.1 and *I*^2^ < 50% was considered no statistical heterogeneity. Pooled summary statistics were calculated with use of a fixed-effect model if heterogeneity was not significant. Meta-regression and subgroup analysis were employed to assess factors responsible for heterogeneity of the primary outcome if heterogeneity was significant. Otherwise, a random effect model was used for unsolvable heterogeneity.

## Results

### Literature search

**Figure 1:**
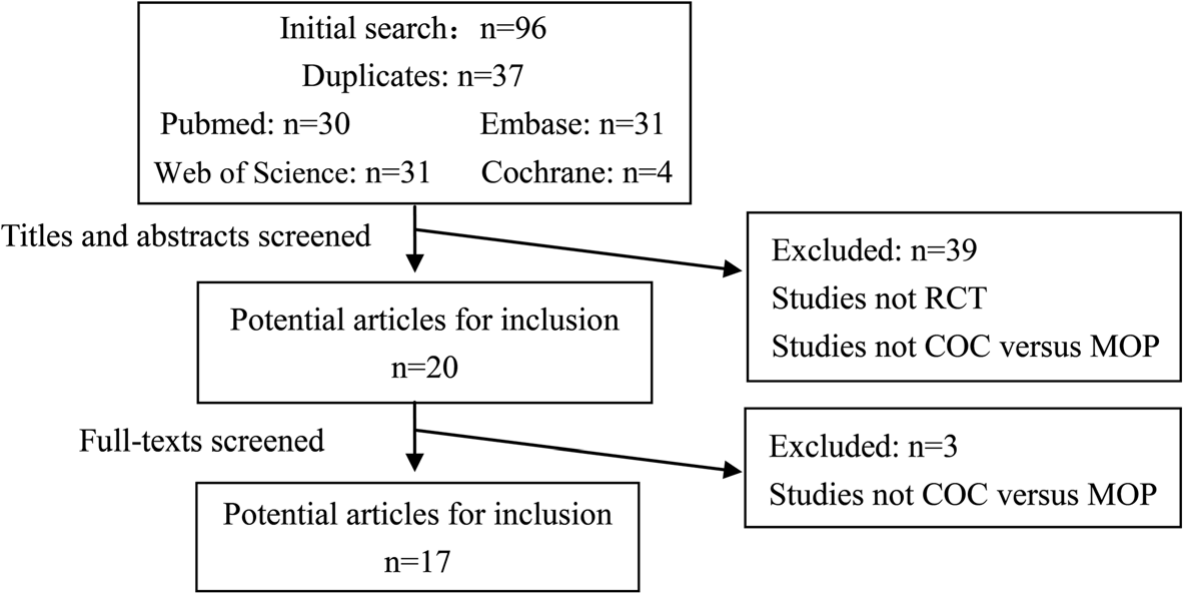
**A flowchart shows the selection of studies for inclusion in the meta-analysis.** *COC* ceramic-on-ceramic, *MOP* metal-on-polyethylene, RCT randomized controlled trial.

Figure 1 summarized the process of identifying eligible studies. Using our search strategy, 96 articles were identified: 30 from Pubmed, 31 from Embase, 31 from Web of science, and 4 from the Cochrane database. Thirty-seven articles were duplicated. The rest 59 articles were selected for screening. From all article titles and abstracts, 20 articles proved potentially eligible and full-text articles were reviewed. Three articles were excluded due to not COC versus MOP. The remaining 17 articles met inclusion criteria, of which 14 articles containing 2 studies were duplicate or updated publications reporting on the same set of patients with primary THA. One study was conducted in multicenter involving 12 duplicates or updated publications [1,3,5,8-16]. The other one was updated involving two articles [4,18]. Finally, 5 studies involving 17 articles were deemed eligible for inclusion. All of the included studies were published in English.

### Study characteristics

The characteristics of five included studies were presented in Table 1. Individual sample sizes were ranging from 61 to 479 patients. Five RCTs involving 897 patients with 974 hips met the above inclusion criteria, of which 601 hips in COC group and 373 in MOP group. One of these five studies was conducted in multicenter [3]. Mean follow-up period of the all five studies was 8.4 years (range from 2 to 15 years). The mean age of patients in the all five studies was 54.5 years.

**Table 1:** **Characteristics of included studies**

**Studies**	**Setting**	**Number of patients**	**Number of hips**		**Mean follow-up (years)**	**Male percentage (%)**		**Range of age (years)**	**Mean age (years)**	
			**COC**	**MOP**		**COC**	**MOP**		**COC**	**MOP**
Bascarevic et al. 2010 [6]	Serbia	150	82	75	4.2	21	31	<65	53.9	55.6
D’Antonio et al. 2012 [1]	USA	479	349	165	10.3	65	60	Unknown	53	53
Nikolaou et al. 2012 [17]	Canada	91	68	34	5	53	50	19–64	53.8	52.0
Vendiittoli et al. 2007 [18]	Canada	116	71	69	12.3	42	55	18–70	54.9	56.8
Zhou et al. 2006 [7]	Australia	61	31	30	2	32	53	46–87	66	68

*COC* ceramic-on-ceramic, *MOP* metal-on-polyethylene.

### Study quality

Study quality assessment of included studies was presented in Table 2. The PEDro scores showed that all of the included five RCTs scored more than 6 and had high methodological quality. All of the five studies used the randomized method. Only one study had a follow-up rate of less than 85% [1]. Four of the five RCTs performed intention-to-treat analysis. None of any study performed therapist blinding because it is difficult in surgical studies.

**Table 2:** **PEDro scores of included studies**

**First author, year**	**PEDro criteria**											**Total**
	**1**	**2**	**3**	**4**	**5**	**6**	**7**	**8**	**9**	**10**	**11**	
Bascarevic et al. 2010 [6]	Y	Y	N	Y	N	N	N	Y	Y	Y	Y	6
D’Antonio et al. 2012 [1]	Y	Y	N	Y	Y	N	Y	N	Y	Y	Y	7
Nikolaou et al. 2012 [17]	Y	Y	Y	Y	N	N	Y	Y	N	Y	Y	7
Vendiittoli et al. 2007 [18]	Y	Y	Y	Y	Y	N	N	Y	Y	Y	Y	8
Zhou et al. 2006 [7]	N	Y	N	Y	N	N	N	Y	Y	Y	Y	6

PEDro criteria: (1) eligibility criteria, (2) random allocation, (3) concealed allocation, (4) baseline comparability, (5) participant blinding, (6) therapist blinding, (7) assessor blinding, (8) >85% follow-up, (9) intention-to-treat analysis, (10) between-groups statistical comparison for at least one key outcome, and (11) point estimates and variability measures for at least one key outcome.

*Y* yes, *N* no.

### Hip function

**Figure 2:**
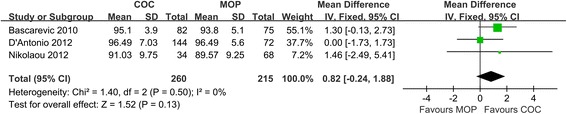
**Forest plot for meta-analysis of Harris hip score.** *COC* ceramic-on-ceramic, *MOP* metal-on-polyethylene.

Three studies (*n* = 475 hips) provided sufficient information about hip functional outcome. No significant difference was found between COC and MOP in postoperative HHS (MD = 0.82; 95% CI, range, −0.24–1.88; *P* = 0.13; homogeneity, *P* = 0.50, *I*^2^ = 0%) (Figure 2). It suggests that the hip function is similar between the two groups.

### Revision

**Figure 3:**
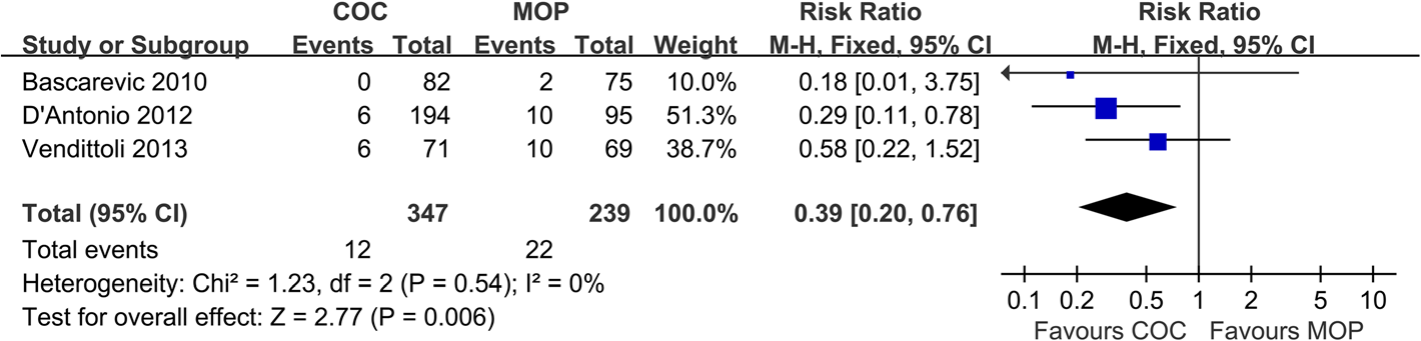
**Forest plot for meta-analysis of revision.** *COC* ceramic-on-ceramic, *MOP* metal-on-polyethylene.

Three studies (*n* = 586 hips) provided detailed information on revision. Figure 3 showed the results of pooled statistical analyses. COC significantly decreased revision rate compared with MOP (3.5% versus 9.2%, respectively; RR = 0.39; 95% CI, range, 0.20–0.76; *P* < 0.01; homogeneity, *P* = 0.54, *I*^2^ = 0%). That means COC was better than MOP on revision.

### Osteolysis and radiolucent line

**Figure 4:**
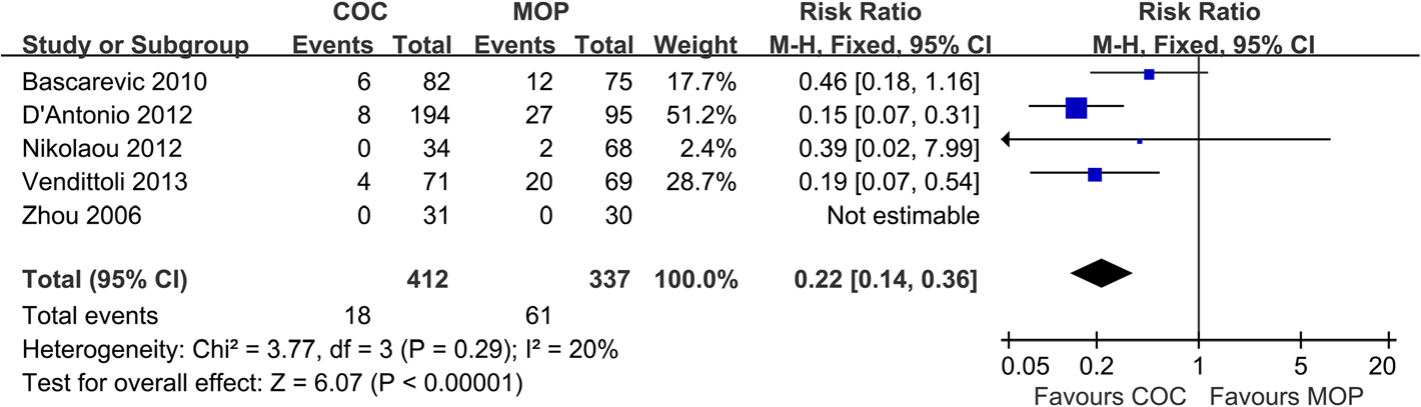
**Forest plot for meta-analysis of osteolysis and radiolucent line.** *COC* ceramic-on-ceramic, *MOP* metal-on-polyethylene.

Pooled analysis of all the five studies (*n* = 749 hips) revealed that COC significantly decreased the incidence of osteolysis and radiolucent line in comparison to MOP (4.4% versus 18.1%, respectively; RR = 0.22; 95% CI, range, 0.14–0.36; *P* < 0.01; homogeneity, *P* = 0.29, *I*^2^ = 20%) (Figure 4). The result is in favor of COC.

### Aseptic loosening

**Figure 5:**
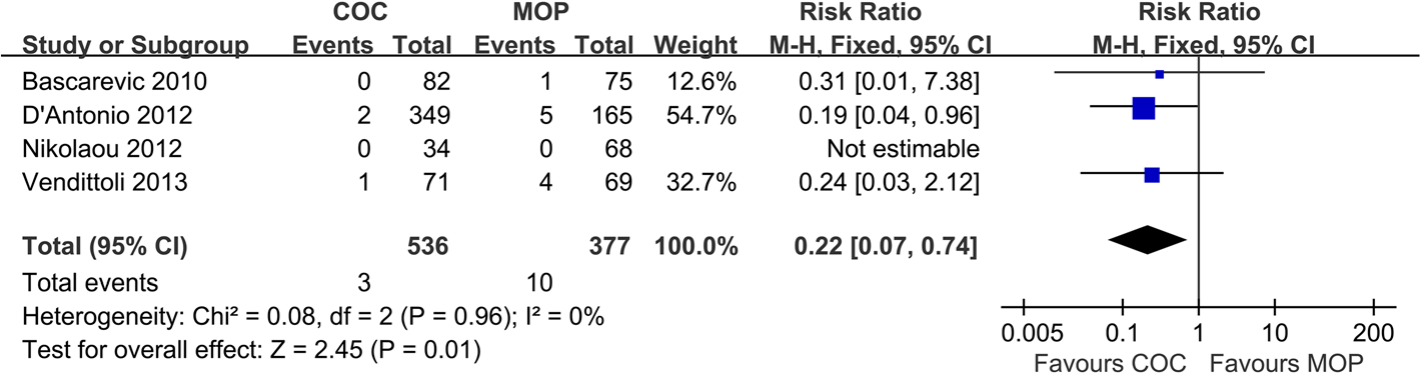
**Forest plot for meta-analysis of aseptic loosening.** *COC* ceramic-on-ceramic, *MOP* metal-on-polyethylene.

Pooled analysis of four studies (*n* = 913 hips) revealed COC significantly decreased the risk of aseptic loosening in comparison to MOP (0.6% versus 2.7%, respectively; RR = 0.22; 95% CI, range, 0.07–0.74; *P* = 0.01; homogeneity, *P* = 0.96, *I*^2^ = 0%) (Figure 5). It shows that COC is superior to MOP on aseptic loosening.

### Squeaking

**Figure 6:**
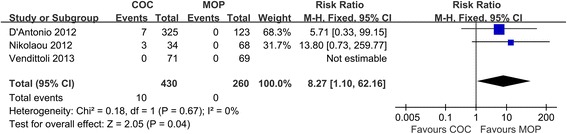
**Forest plot for meta-analysis of squeaking.** *COC* ceramic-on-ceramic, *MOP* metal-on-polyethylene.

Three studies (*n* = 690 hips) provided detailed information on squeaking. Pooled analysis of these studies revealed COC significantly increased the risk of squeaking in comparison to MOP (2.3% versus 0%, respectively; RR = 8.27; 95% CI, range, 1.10–62.16; *P* = 0.04; homogeneity, *P* = 0.67, *I*^2^ = 0%) (Figure 6). All squeaking occurred in COC and none in MOP.

### Implant fracture

**Figure 7:**
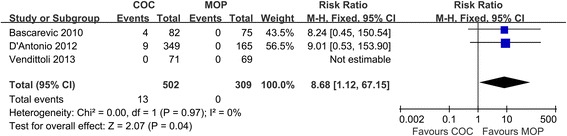
**Forest plot for meta-analysis of implant fracture.** *COC* ceramic-on-ceramic, *MOP* metal-on-polyethylene.

Pooled analysis of three studies (*n* = 811 hips) revealed that COC significantly increased intraoperative implant fracture rate compared with MOP (2.6% versus 0%, respectively; RR = 8.68; 95% CI, range, 1.12–67.15; *P* = 0.04; homogeneity, *P* = 0.97, *I*^2^ = 0%) (Figure 7). Three studies provided detailed information on postoperative implant fracture in which only one case of postoperative implant fracture in COC group has been reported [17]. In general, the result is in favor of MOP.

### Dislocation

**Figure 8:**
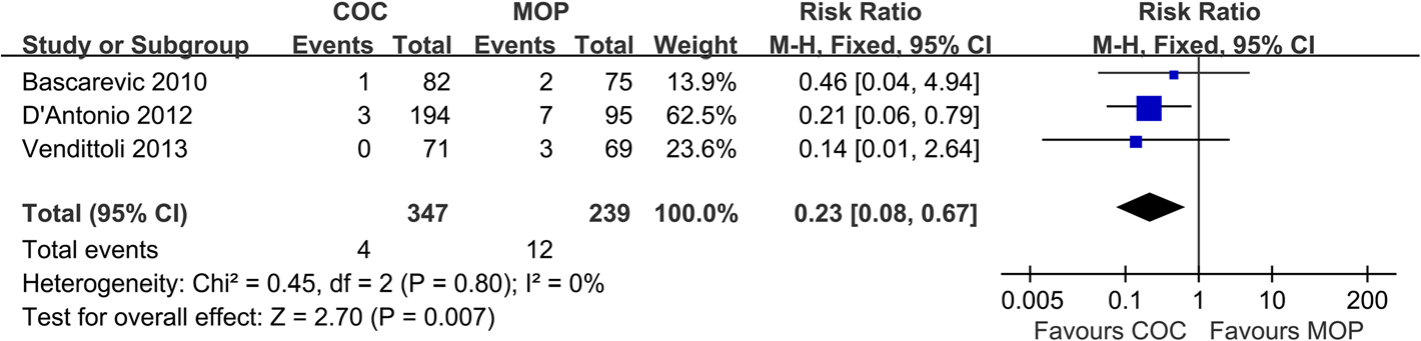
**Forest plot for meta-analysis of dislocation.** *COC* ceramic-on-ceramic, *MOP* metal-on-polyethylene.

The forest plot of three studies (*n* = 586 hips) indicated that COC significantly decreased dislocation rate in comparison to MOP group (1.2% versus 5.0%, respectively; RR = 0.23; 95% CI, range, 0.08–0.67; *P* < 0.01; homogeneity, *P* = 0.80, *I*^2^ = 0%) (Figure 8). The result is in favor of COC.

### Other complications

Pooled analysis of studies did not reveal any difference in the risks of deep infection and heterotopic ossification between COC and MOP groups (*P* > 0.05).

## Discussion

Debates are ongoing regarding the optimal bearing surface for THA, especially in young and active patients [2,7,17,18]. Some researchers reported that COC was superior to MOP clinically [4,5] while the others concluded there were no significant difference between them [6,7,17]. The results of the current meta-analysis revealed that COC significantly decreased the risks of osteolysis and radiolucent line, aseptic loosening, and revision as well as dislocation. But it also had the significantly higher risks of squeaking and intraoperative implant (ceramic) fracture, which the MOP THA did not have at all. For postoperative hip function, deep infection, and heterotopic ossification, the outcomes were similar between COC and MOP.

It has been recognized that one of the leading causes of implant failure in THA is aseptic loosening due to osteolysis [17]. There is a general acceptance that aseptic osteolysis occurs because of chronic inflammatory response to implant-derived wear debris which leads to subsequent bone resorption [8]. The most common wear debris mode is polyethylene wear [10]. Although new-generation highly cross-linked polyethylene (HXLPE) significantly reduces wear in comparison to conventional ultra-high molecular weight polyethylene (UHMWPE), the polyethylene wear and its debris still cannot be ignored [17]. A new meta-analysis which include eight studies involving 735 patients reported that HXLPE significantly reduced radiological wear but not osteolysis or wear-related revision in comparison to conventional UHMWPE at midterm follow-up periods [21]. For the aforementioned reason, ceramic liner was reintroduced as an alternative due to its excellent wear resistance. Literature review showed that osteolysis was rarely observed at a wear rate of <100 μm/year [22]. Wear rate >150 μm/year put the prosthesis at risk of aseptic loosening. A 25-year survivorship of prosthesis with a wear rate <100 μm/year exceeded 90%, but a 20-year survivorship of prosthesis with a wear rate >200 μm/year was below 30%, and none survived 25 years [23]. In current meta-analysis, two of the five included studies reported the wear rate of COC and MOP. The wear rate of COC was below detection and 6.7 μm/year while that of MOP was 190 μm/year and 107.7 μm/year, respectively [4,17]. It is obvious that the wear rate of COC was much lower than that of MOP. The difference could determine prosthesis survivorship according to the findings mentioned above, and this was further demonstrated by clinical investigation. Mesko et al. [16] reported that the survivorship for COC was significantly higher than that for MOP at 10 years. This meta-analysis demonstrated that COC significantly decreased the risks of osteolysis and radiolucent line, aseptic loosening, and revision in comparison to MOP, which implies that COC prosthesis has better and longer survivorship. Although previous meta-analysis reports that MOP revealed higher survival rates than COC, just as what is previously mentioned, the data are not convincing. It is largely based upon observational studies, it follows a random effects model, and the *P*-value is marginal (*P* = 0.05). By contrast, our data are more objective, reliable, and persuasive.

Ceramic is a brittle material. Ceramic fracture, including intraoperative and postoperative ceramic fracture, remains one of the most important complications that should be considered. This meta-analysis showed that all the intraoperative implant fractures occurred in COC group. Intraoperative implant fracture is the unique complication of COC. Intraoperative ceramic fracture usually occurs during the process of insertion of the ceramic liner into the metal acetabular cup due to improperly seated liners or cup deformation which can result from tapping the edge of the cup in attempt to adjust its position, especially when impacting it into a hard bone [24,25]. Hence, the insertion of ceramic liner into the metal acetabular cup requires great attention and precision. Some studies highlighted the complication of postoperative ceramic fracture in COC. Nevertheless, there was only one case that reported postoperative ceramic fracture in three of the included studies involving 347 hips in the current meta-analysis. Although early experience with ceramic bearing surfaces met with disappointing results due to ceramic fracture resulting from insufficient purity, low density, a coarsely grained microstructure, and implant design [5,15], hot isostatic thermal pressing of the relative new third-generation ceramic has made it highly resistant to breakage [6,15]. Ceramic used in all of the five included studies in this meta-analysis was the third-generation product, and the rate of intraoperative ceramic fracture was 2.6%. The new fourth-generation Delta ceramic which has a smaller grain size was developed with improved wear resistance while reducing the risk of ceramic fracture and achieved excellent clinical results [26]. With the development of material manufacture, the incidence of ceramic fracture declined progressively [15].

COC significantly increased the risk of squeaking comparing with MOP, and all squeaking occurred in COC THAs. The reported incidence of squeaking after COC THA varies between 0.3% and 20.9% [27,28]. Our result is 2.3% which is consistent with literatures. Squeaking can be intolerable enough for some patients to seek revision [16,29]. The etiology of squeaking is believed to be multifactorial and related to surgical technique, implant design, and patient factors [1,5]. Possible mechanisms include microseparation associated with impingement, stripe wear, edge loading, shortened femoral necks, and cup malposition [28,30,31]. Squeaking could be avoided to some degree, especially due to surgical technique errors. It can be prevented through medialization of the acetabular cup and avoidance of the use of shortened femoral necks which may result in neck rim impingement [30].

The incidence of dislocation rate was 1.2% for COC and 5.0% for MOP, which is significantly different. It is possible that the dislocation was related to the head size rather than the bearing surface used [32]. Studies have been shown that large diameter femoral heads have a lower dislocation rate [32-34]. It should be noted that most of the COC received 32 mm or larger femoral heads while most of the MOP received 28 mm or smaller femoral heads [4,15,18]. Basis on present manufacture technology, polyethylene could not be done as thin as ceramic. Therefore, COC have the opportunity to match larger femoral heads in the same size of the acetabular cup. That means that most of the patients in COC received a larger femoral head than did the patients in MOP. This may attribute to low dislocation rate of COC comparing with MOP in this meta-analysis.

All of the five included studies have high methodological quality. And all the RCTs in this meta-analysis recruited relative young patients with mean age of 54.5 years old. Young patients are expected to have higher demands on THAs, not only because they are more active but also because they have a longer life expectancy [2]. However, this meta-analysis has several limitations. Firstly, the average follow-up period of the all five included RCTs was 8.4 years, which is not long enough for a better assessment of prosthesis survivorship. Secondly, this meta-analysis included only five studies involving 897 patients with 974 hips. The sample size is not large enough. Thirdly, this study was limited to articles published in English. There might exists publication and language bias. But the small number of included studies limits our ability to assess publication and language bias. Fourthly, different types of polyethylene were used in the five included studies. Two studies [1,4] used conventional UHMWPE while another two [6,7] used HXLPE. The rest one studied both UHMWPE and HXLPE [17]. This could be a confounding factor in this meta-analysis.

## Conclusion

This meta-analysis of RCTs suggests that COC decreased the risks of revision, osteolysis, aseptic loosening and dislocation, but it also increased the risks of squeaking and intraoperative ceramic fracture comparing with MOP. Generally, our findings support the use of COC bearing surface.
